# Risk Indicators for Early Childhood Caries in South Africa: Protocol for a Systematic Review

**DOI:** 10.2196/26701

**Published:** 2021-06-24

**Authors:** Faheema Kimmie-Dhansay, Robert Barrie, Tina Roberts, Sudeshni Naidoo

**Affiliations:** 1 Research and Higher Degrees Faculty of Dentistry University of the Western Cape Cape Town South Africa

**Keywords:** dmft, infant, risk factors, dental caries, South Africa, early childhood caries

## Abstract

**Background:**

Early childhood caries (ECC) is a common disorder characterized by the presence of one or more decayed (non-cavitated or cavitated lesions), missing (due to caries), or filled tooth surfaces of primary teeth in children 71 months old or younger. South Africa has a diverse population in terms of culture, education, income, and occupation. This diversity is due to the consequences of historical racial discrimination, poverty, unemployment, lack of accessibility to health services, and quality of education. These factors make South Africa unique, and the disease and risk profiles for this country differ from those of other countries at similar stages of development. For these reasons, it is important to identify the unique maternal and infant risk factors for ECC in the South African context.

**Objective:**

The purpose of this study is to determine the risk factors associated with the incidence and prevalence of ECC in South Africa in children under the age of 6 years.

**Methods:**

All cross-sectional and cohort studies documenting risk factors associated with the prevalence and incidence of dental disease and severity (decayed, missing, and filled scores) will be included. We will search 7 databases for eligible studies, and those included will be based on prespecified inclusion criteria. Only studies conducted with South African children who are aged 6 years and younger in which dental caries risk factors are documented will be included. There is no restriction on the time or language of publication. Included articles will be scrutinized for quality by using a risk of bias tool developed by the Joanna Briggs Institute. The results will be presented narratively, and if possible, a meta-analysis will be performed.

**Results:**

The literature search was conducted in November 2020.

**Conclusions:**

The results of this study will provide a framework to inform medical and dental personnel to highlight mothers and infants at risk of developing ECC.

**Trial Registration:**

PROSPERO International Prospective Register of Systematic Reviews CRD42020216455; https://www.crd.york.ac.uk/prospero/display_record.php?RecordID=216455

**International Registered Report Identifier (IRRID):**

DERR1-10.2196/26701

## Introduction

Noncommunicable diseases (NCDs), including early childhood caries (ECC), contribute to global public health challenges. Of the world’s population, 26% are under the age of 16 years [1], and approximately 2.1 billion children are affected by NCDs [2]. Untreated dental caries in primary teeth is the tenth most prevalent health condition worldwide [3].

ECC is a common disorder characterized by the presence of one or more decayed (noncavitated or cavitated lesions), missing (due to caries), or filled tooth surfaces of primary teeth in children 71 months old or younger. Severe ECC is either any smooth tooth surface decay in children under 3 years old or one or more cavitated, missing (due to caries), or filled smooth tooth surfaces in the primary maxillary anterior teeth in children between the ages of 3 and 5 years [4].

Young children depend on either their parents or caregivers for their health needs and general well-being. For this reason, the responsibility of preventing NCDs such as ECC in this age group lies exclusively with their carers. There are several theoretical frameworks proposing the evolution of ECC [5-8] and an equal number of strategies aimed at preventing the disorder [9-12]. However, the battle against ECC continues.

In the past few decades, several researchers have investigated the etiology of ECC. Like dental caries, ECC occurs as an interplay of at least 4 factors: cariogenic bacteria, fermentable carbohydrates, a susceptible tooth surface, and time. Traditionally, *Streptococcus mutans* (*S. mutans*) has been implicated as the key microorganism of dental caries. Recent studies based on DNA and RNA extracted from carious lesions have revealed that *S. mutans* forms a small part of a diverse biome that interacts synergistically to initiate and enlarge a carious lesion [13]. Oral bacteria can metabolize fermentable carbohydrates such as monosaccharides resulting in an increased production of acids capable of demineralizing dental hard tissue, especially enamel, over a period of time [13]. Apart from the known causative factors, several risk factors are associated with ECC.

Both maternal and infant risk factors have been implicated in the initiation and evolution of ECC. These include socioeconomic status, level of parental education, maternal age, caregiver depression, Vitamin D deficiency, and immigration status. Dental epidemiologists have explored the relationship between ECC and various risk factors for many decades. Despite their efforts, there has been little agreement on the findings, and ECC remains a global challenge affecting countless children.

South Africa has a diverse population in terms of culture, education, income, and occupation. This diversity is due to the consequences of historical racial discrimination, poverty, unemployment, lack of accessibility to health services, and quality of education. Furthermore, there is a distinct divide in the socioeconomic standing of the South African population that results in inequalities in essential health and oral health care [14]. These factors make South Africa unique, and the disease and risk profiles may differ from those of other countries at similar stages of development. In South Africa, children with ECC treated in public state facilities often present for multiple extractions under dental general anesthesia [15]. Apart from the financial costs and long waiting lists, repeated use of general anesthesia in young children poses inherent risks [15].

The aim of this study is to identify the unique maternal and infant risk factors for ECC in a South African context. The authors are confident that the outcome of the present investigation will raise awareness among caregivers, oral health professionals, general practitioners, and other stakeholders [16].

## Methods

This systematic review will collect and synthesize data to determine the maternal and infant risk factors for ECC in South Africa. This protocol will be conducted according to the PRISMA-P (Preferred Reporting Items for Systematic reviews and Meta-Analyses for Protocols) guidelines [17]. The search strategy will only be used to identify published studies conducted within South Africa. An initial search was used to identify suitable articles. A search strategy will be developed for each database. The words in the title and abstracts will be screened to determine the initial suitability of the articles. All references of included articles will be perused for more suitable titles. The search strategy will include the following terms: (((“South Africa”) AND (children OR “peri-natal” OR paediatric OR pediatric OR neonatal OR infant)) AND (“risk factor*” OR risk or factor*)) AND (“early childhood caries” OR caries OR decay OR dmft OR dental OR oral OR PUFA).

### Eligibility Criteria

Cross-sectional and cohort studies that report on the maternal and infant risk factors for ECC in South Africa will be included in the review. There will be no limitations pertaining to the date of publication. Studies published in all South African official languages will be included. Non-English articles will be translated by the Department of Foreign Languages at the University of the Western Cape or a reputable translation company. To validate the translations, this paper will cross-reference the original article with the English abstract (which is usually available online), and reverse translations will be conducted to ensure its validity.

### Information Sources

#### Search Strategy

Two independent reviewers will search the identified search engines to locate both published and unpublished studies.

#### Study Selection and Data Management

Two reviewers will select studies for inclusion based on the inclusion and exclusion criteria. An initial search of PUBMED was undertaken to identify articles pertaining to the maternal and infant risk factors for ECC in South Africa. The text words contained in the titles and abstracts of relevant articles and the index terms will be used to develop a full search strategy for PubMed/Medline, Cochrane, Scopus, Academic Search Complete, Dentistry and Oral Science sources, CINAHL, and Science Direct. The search strategy, including all identified keywords and index terms, will be adapted for each database or information source.

Commentaries or letters and other grey literature will be excluded from this review.

Secondary searching (PEARLing) will be conducted (PEARLing is a search strategy where the reference lists of all the studies, whether included or excluded, are identified for possible inclusion). Manual searching will not be conducted due to the difficulty in replicating this method.

The literature search will be recorded in a data capture sheet (Multimedia Appendix 1) that will include the source, the date of search, the number of hits, and a reference link to the publications. This will be carried out after duplications have been resolved. Study selection will be blinded, and any disagreements will be resolved by discussion. A data extraction tool (Multimedia Appendix 2) will be used to guide reviewers on the data that would need to be extracted from selected articles. The extracted data will be recorded in an Excel spreadsheet and will include publication details, the setting of the research study, age of the population, gender, periodontal disease clinical determinants, number of cases, and total sample size. Rayyan [18] will be used to resolve duplications and record all reviewer decisions.

All studies will be included, regardless of methodological quality. The quality assessment of studies will be performed using the Joanna Briggs Institute Critical Appraisal Checklist for Studies [19]. There will be 2 reviewers involved in the quality assessment, and disagreements will be resolved through discussion.

A meta-analyses of studies with similar comparisons reporting the same outcomes will be conducted. This paper will use a random-effects model if there are 4 or more studies. This paper will report the results from studies not suitable for inclusion in a meta-analysis. There will be no I^2^ cut-off point to assess heterogeneity. Meta-analysis will be performed using STATA 15.

## Results

This review will be conducted from January 2021 to June 2021. The Prospero registration number is CRD42020216455**.** Two reviewers will be blinded at each stage of the process. Regular team meetings will be held to settle any disputes or differences between the reviewers’ inclusions. This will serve to enhance the reliability and validity of the review process. All discussions will be recorded as evidence. There will be no conflict of interest within the team.

### Study Selection

Papers will be uploaded into Rayyan [18] and screened in 2 stages. Two review authors will independently assess all the titles and abstracts of the identified studies against the inclusion criteria. For each study appearing to meet the inclusion criteria or where there is insufficient information to make a clear decision, the full text will be obtained, and 2 review authors will independently assess it to establish whether the study meets the inclusion criteria. Where agreement is not achieved, a third review author will be contacted. The searching process will include all eligible studies published before November 15, 2020. All eligible studies will be included, and authors will be contacted if any further clarification is needed.

After reading full-text articles, those that do not meet the inclusion criteria will be discarded. Details of the excluded studies and reasons for their exclusion will be documented in a “Characteristics of excluded studies” table. The reference list of all included studies will be investigated for further eligible studies.

### Data Extraction and Management

#### Data Extraction and Recording Process

Two review authors will independently extract the data from the studies using a predefined data extraction form (initially piloted on a small sample of studies). Any discrepancies will be resolved through consultation with a third review author. If any information from the studies is unclear or missing, the authors of the original papers (where feasible) will be contacted for further information.

Study information data include author, title, year of publication, study design, and year that study was conducted.

Participant level data including maternal risks and infant risks, such as socio-demographic factors; dietary factors; oral hygiene factors; factors related to bottle or breast-feeding; oral bacterial flora; and other factors will be recorded.

#### Availability of Data and Materials

If study articles are not obtainable, a librarian will be consulted, and if the study is still not obtainable, the study will not be included in the qualitative or quantitative analysis.

#### Study Quality and Risk of Bias Assessment

The quality assessment of studies will be performed using the Joanna Briggs Institute Critical Appraisal Checklist for Studies reporting Prevalence Data [20].

#### Analysis of Study Findings

A meta-analysis of findings may be performed.

### Results to Date

This protocol was registered with PROSPERO in November 2020, and the completed electronic searches were performed by November 15, 2020. The original search yielded 1366 articles. This study aims to highlight the risk factors associated with the incidence or prevalence of ECC in South Africa in children under the age of 6 years. The findings of this study will be used to provide stakeholders with the necessary tools to educate policymakers on the best framework to prevent or limit this disease and future NCDs in childhood and adolescence.

## Discussion

### Overview

The study aims to determine the maternal and infant risk factors that can contribute to the incidence or prevalence of dental caries in children under the age of 6 years in South Africa. Current evidence suggests that *S. mutans*, enamel defects, and presence of dental caries are responsible for the incidence of dental caries in this population [21]. However, with South Africa being the most unequal county in the world with a Gini index of 63 [22], it is not surprising that there may be other risk factors such as sociodemographic factors that could be playing a role in the development of this disease.

### Conclusions

There is a paucity of information on maternal and infant risk factors associated with the incidence or prevalence of ECC in South Africa. There is a need to synthesize the existing data to highlight risk factors that could limit the scourge of this preventable disease.

## Appendix

Multimedia Appendix 1 Search strategy.

Multimedia Appendix 2 Data capture sheet.

## Abbreviations

ECC: early childhood caries
NCD: noncommunicable disease
PRISMA-P: Preferred Reporting Items for Systematic reviews and Meta-Analyses for Protocols
