# Supplementary material for: Risk Indicators for Early Childhood Caries in South Africa: Protocol for a Systematic Review
Source: JMIR Res Protoc. 2021 Jun 24;10(6):e26701. doi: 10.2196/26701 (PMC8386354; doi:10.2196/26701)
Supplement: Multimedia Appendix 1 [file resprot_v10i6e26701_app1.pdf]

Annexure 1

Search Strategy

Date of Search	Database source	Reference link	Search Strategy	Number of hits
