# Supplementary material for: Risk Indicators for Early Childhood Caries in South Africa: Protocol for a Systematic Review
Source: JMIR Res Protoc. 2021 Jun 24;10(6):e26701. doi: 10.2196/26701 (PMC8386354; doi:10.2196/26701)
Supplement: Multimedia Appendix 2 [file resprot_v10i6e26701_app2.pdf]

Annexure 2

Data capture sheet

Article title	Author	year	Socio-demographic factors	Dietary Factors	Oral Hygiene factors	Factors related to bottle or breast-feeding	Oral bacterial flora	Other factors
